# The Principles of Management of Visceroptosis

**Published:** 1909-06-12

**Authors:** 


### Medicine.

### THE PRINCIPLES OF MANAGEMENT OF VISCEROPTOSIS.

The ætiology of visceroptosis is embraced under three main headings, namely: (1) congenital defects; (2) pregnancy; and (3) prolonged impairment of general nutrition. Of these, the third is by far the most important. Under congenital defects should be grouped mainly those cases in which the symptoms arise during early life, and in which no other cause can be determined. Repeated pregnancies at short intervals lead to relaxation both of the abdominal walls and of the pelvic floor.

Malnutrition, or rather impairment of general nutrition, may result from any unhygienic modes of life, whether in the direction of excess of eating with deficiency in exercise amongst the well-to-do, or in the direction of close confinement, prolonged labour in factories, deficient food, and so forth amongst the working classes. Corsets, falls, and direct blows in the regions of the various abdominal organs probably have no effect on their position unless one of the causes mentioned above has first existed.

Should any chronic toxæmia have become established a vicious circle at once becomes active. The nervous disturbances affect digestion deleteriously, whilst stagnation in the digestive tract increases the poisoning of the circulation and so hastens degeneration of the nervous system and muscles. Each condition thus reacts upon the other to exaggerate it.

For purposes of management Dr. Halsey groups the cases according to symptoms as follows: (1) Those in whom the nervous symptoms are the most prominent; (2) those who complain most of digestive disturbances; (3) those in whom organic displacements account for distress.

He groups the treatment under six main headings, namely those of hygiene, rest, passive and active exercise, mechanical support, drugs, and operation.

Hygiene includes regular hours for sleep, bathing, eating; and life in the open air. These are all essential in every case of visceroptosis. The clothing about the body should be worn loose. The custom of wearing about the waist tightly constricting bands, belts, or strings from which the skirts or trousers are supported must be discontinued. All weight of the clothing should come upon the shoulders; otherwise the good arising from support of the lower segment of the abdomen will be counteracted by the downward pull of the constricted upper segment.

Rest may require prolonged application in isolation, as in the Weir-Mitchell treatment of neurasthenia. The diet should consist of all classes of food taken at frequent intervals in small amounts. Rapid improvement of nutrition follows the use of such foods properly prepared so as to be easily absorbed from the weakened digestive apparatus. Fluid should be used in large amounts, but it should be administered at such times as will ensure its not interfering with food digestion. Rectal enemata of normal saline solution may sometimes be necessary either to cleanse the bowel or to increase fluid absorption.

Exercise should at first comprise passive massage and resistent movements. Later the different muscles may be exercised actively. Particular attention should be directed to increased functional use of the abdominal muscles; movements in imitation of rowing, and of mowing with a scythe, are useful here. Under no circumstances should they be carried to the point of fatigue, though a brief rest after each period of exercise will accelerate the gain. As soon as may be the exercises should be carried on in the open air, and the more enjoyable they can be made the better.

Mechanical support is directed to the segment of the abdomen below the umbilicus. Although radiographs show no replacement of the hollow viscera, the effectual support of the lower segment does relieve the symptoms and aid materially in improving the nutrition. Improvement of nutrition may accomplish only the relief of the symptoms, yet in some cases it effects complete fixation, after which the mechanical support can be dispensed with.

It is absolutely essential that the mechanical method selected, whatever else it does, should hold back the lower abdominal segment; otherwise it will afford no benefit. It may be noted that support applied in this way relieves symptoms even when there is no prominence of the lower segment, and without raising the lower border of the stomach. The degree of pressure exerted probably diminishes chronic congestion and also diminishes the strain on ligaments and adhesions. Mechanical support, except in cases of separation or excessive relaxation of the abdominal muscles, should be employed only as a temporary expedient whilst the other means of improvement are gradually taking effect.

Such drugs should be employed as will improve the tone and meet the symptoms at the moment. In nervous cases it may be necessary to give sedatives or hypnotics for a while, but they should be used only as a temporary expedient, and never for prolonged periods. Glycero-phosphates, iron, arsenic, ergot, cocoa, strychnine may all be employed at different times in varying doses according to the case. A very useful all-round prescription is: Syr. Hypophosph. Co. (B.P.C.), ʒj. t.d.s.p.c. ex aquâ ℥j.; Cathartics should rarely be resorted to—they are used far too often.

When there are definite symptoms from displacement of an organ, and all the above lines of treatment have failed, then operation must be resorted to. The pelvic organs should be replaced, and repaired if there are lacerations or prolapse. The kidney may be fixed if it is clear that there is an intermittent blocking of the outlet—though it is important to avoid even the suggestion of an operation when the kidney is merely felt to be movable, without there being definite local symptoms. The liver or the spleen may occasionally need suturing in place.

